# Supplementary material for: Too Few, Too Many, or Just Right? Optimizing Sample Sizes for Population‐Level Inferences in Animal Tracking Projects
Source: Ecol Evol. 2026 May 31;16(6):e73755. doi: 10.1002/ece3.73755 (PMC13239661; doi:10.1002/ece3.73755)
Supplement: Supplementary file 3 — Data S3: ece373755‐sup‐0003‐DataS3.pdf. Figure S3:1 Relative error (%) in home range estimates (AKDE) as a function of population sample size (𝒎), ranging from 2 to 50 simulated African buffalos ( Syncerus caffer ). Each vertical facet corresponds to one of five sampling durations: 2 months, 4 months, 1 year, 3 years, and 9 years. The 1 10 plot shows mean estimates based on a single combination of individuals per 𝑚 (no resampling); estimates in blue are within the error threshold and 11 12 13 14 15 16 17 18 19 in red if estimates fall outside of it. Horizontal dotted lines mark the ±5% error threshold. Figure S3:2 Relative error (%) in home range estimates (AKDE) as a function of population sample size (𝒎), ranging from 2 to 50 simulated Mongolian gazelles ( Procapra gutturosa ). Each vertical facet corresponds to one of five sampling durations: 2 months, 4 months, 1 year, 3 years, and 9 years. The plot shows mean estimates based on a single combination of individuals per 𝑚 (no resampling); estimates in blue are within the error threshold and in red if estimates fall outside of it. Horizontal dotted lines mark the ±5% error threshold. Figure S3:3 Relative error (%) in speed estimates (CTSD) as a function of population sample size (𝒎), ranging from 2 to 50 simulated African buffalos ( Syncerus caffer ). Each vertical facet corresponds to one of five sampling intervals: 4 h, 3 h, 2 h, 1 h, and 30 min. The 20 2 21 plot shows mean estimates based on a single combination of individuals per 𝑚 (no resampling); estimates in blue are within the error threshold and 22 23 24 25 26 27 in red if estimates fall outside of it. Horizontal dotted lines mark the ±5% error threshold. Figure S3:4 Relative error (%) in speed estimates (CTSD) as a function of population sample size (𝒎), ranging from 2 to 50 simulated Mongolian Gazelles ( Procapra gutturosa ). Each vertical facet corresponds to one of five sampling intervals: 4 h, 3 h, 2 h, 1 h, and 30 min. The plot shows mean estimates b [file ECE3-16-e73755-s004.pdf]

Too few, too many, or just right? Optimizing sample sizes for population-level inferences in animal tracking projects

S3. Single combination plots

Home range estimation

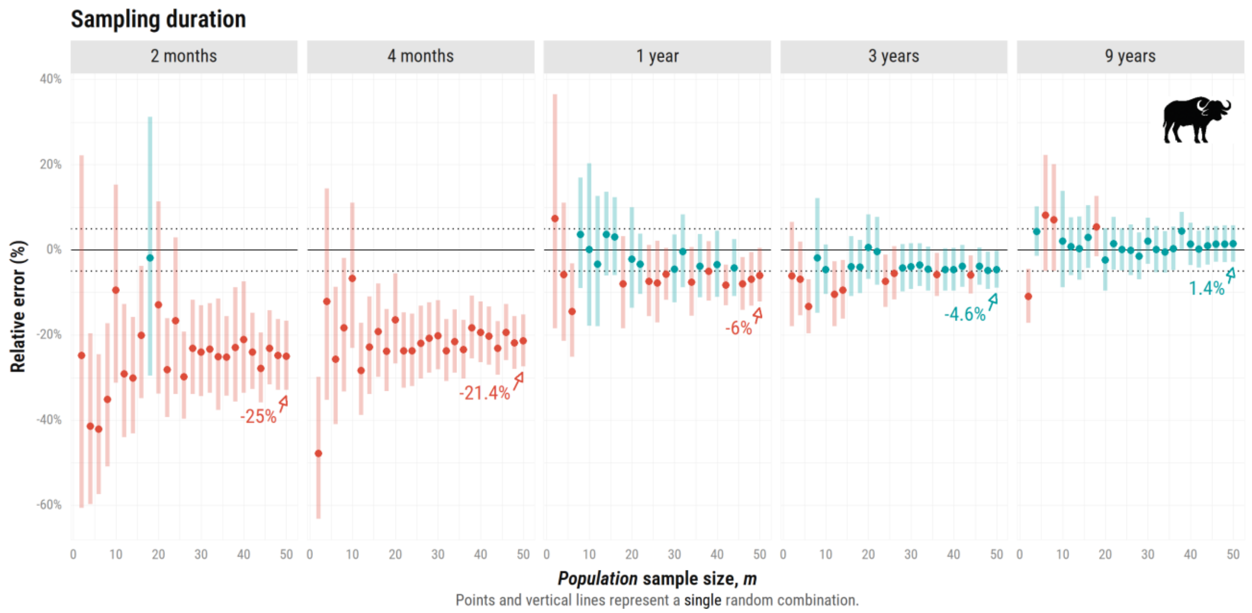

Figure S3.1. Relative error (%) in home range estimates (AKDE) as a function of *population* sample size (m), ranging from 2 to 50 simulated African buffalos (*Syncerus caffer*). Each vertical facet corresponds to one of five sampling durations: 2 months, 4 months, 1 year, 3 years, and 9 years. The

plot shows mean estimates based on a **single** combination of individuals per m (no resampling); estimates in blue are within the error threshold and in red if estimates fall outside of it. Horizontal dotted lines mark the ±5% error threshold.

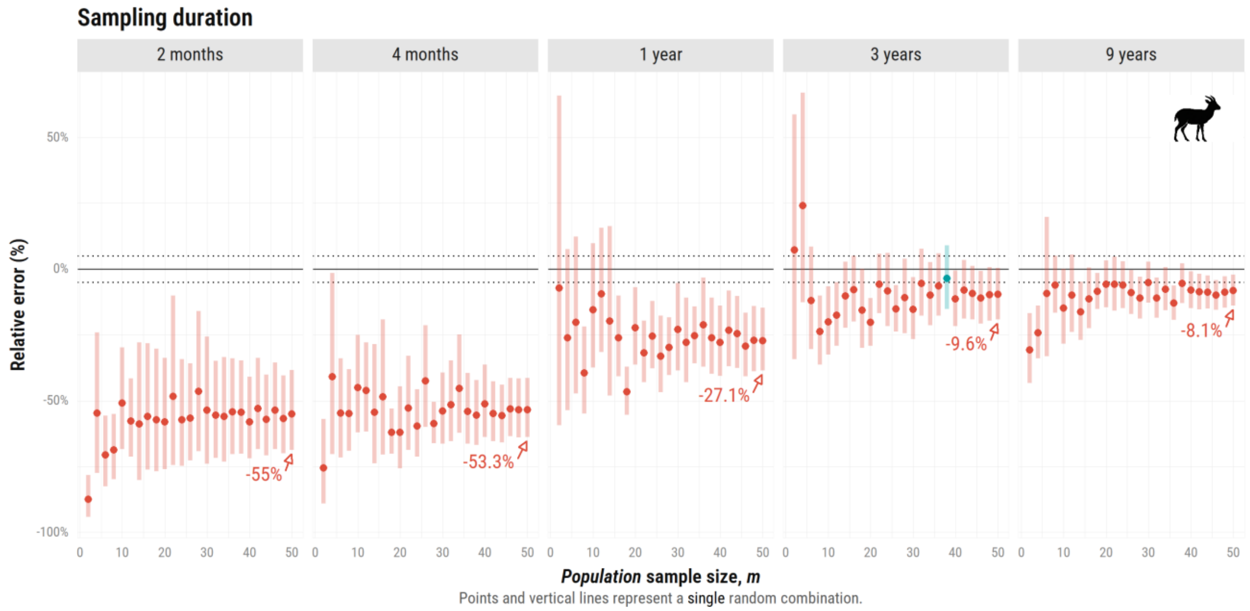

Figure S3.2. Relative error (%) in home range estimates (AKDE) as a function of *population* sample size (m), ranging from 2 to 50 simulated Mongolian gazelles (*Procapra gutturosa*). Each vertical facet corresponds to one of five sampling durations: 2 months, 4 months, 1 year, 3 years, and 9 years. The plot shows mean estimates based on a **single** combination of individuals per m (no resampling); estimates in blue are within the error threshold and in red if estimates fall outside of it. Horizontal dotted lines mark the ±5% error threshold.

Speed & distance estimation

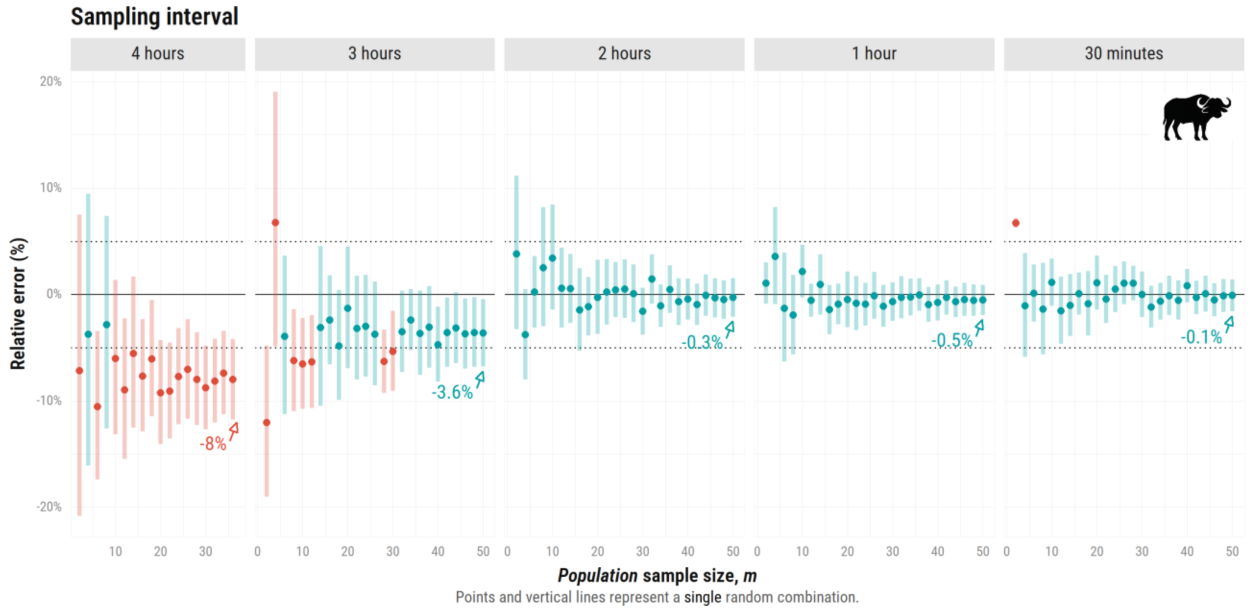

Figure S3.3. Relative error (%) in speed estimates (CTSD) as a function of *population* sample size (m), ranging from 2 to 50 simulated African buffalos (*Syncerus caffer*). Each vertical facet corresponds to one of five sampling intervals: 4 hours, 3 hours, 2 hours, 1 hour, and 30 minutes. The

plot shows mean estimates based on a single combination of individuals per m (no resampling); estimates in blue are within the error threshold and in red if estimates fall outside of it. Horizontal dotted lines mark the ±5% error threshold.

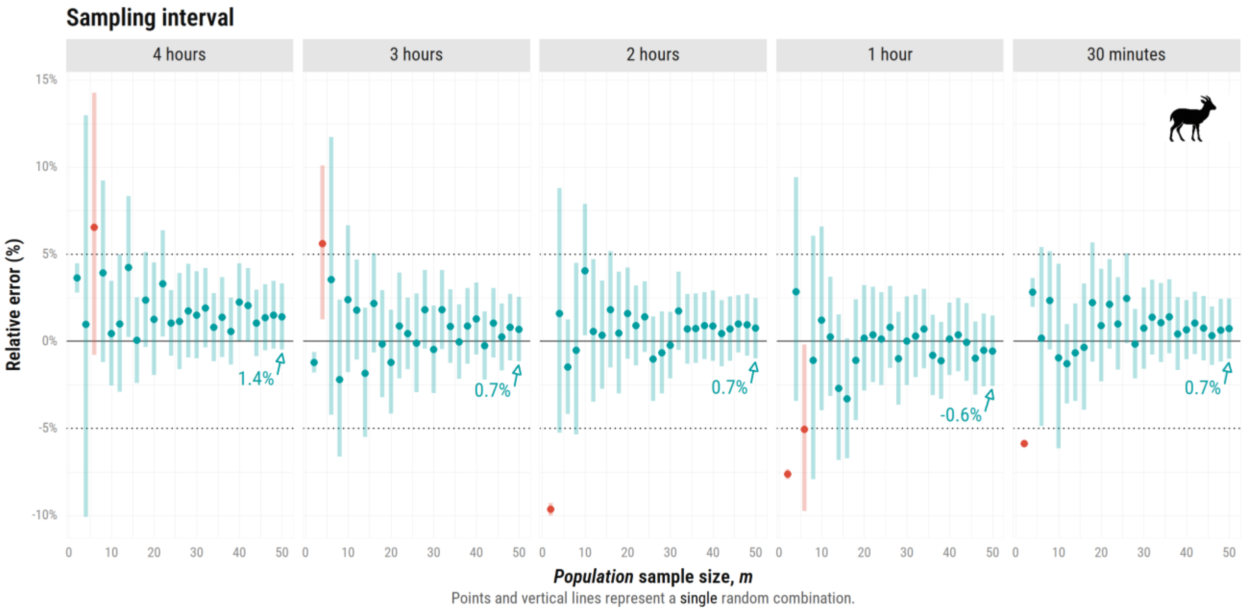

Figure S3.4. Relative error (%) in speed estimates (CTSD) as a function of *population* sample size (m), ranging from 2 to 50 simulated Mongolian Gazelles (*Procapra gutturosa*). Each vertical facet corresponds to one of five sampling intervals: 4 hours, 3 hours, 2 hours, 1 hour, and 30 minutes. The plot shows mean estimates based on a **single** combination of individuals per m (no resampling); estimates in blue are within the error threshold and in red if estimates fall outside of it. Horizontal dotted lines mark the ±5% error threshold.
