# Supplementary material for: Too Few, Too Many, or Just Right? Optimizing Sample Sizes for Population‐Level Inferences in Animal Tracking Projects
Source: Ecol Evol. 2026 May 31;16(6):e73755. doi: 10.1002/ece3.73755 (PMC13239661; doi:10.1002/ece3.73755)
Supplement: Supplementary file 4 — Data S4: ece373755‐sup‐0004‐DataS4.pdf. Figure S4:1 Relative error (%) in home range area estimates across different sampling durations for simulated African buffalos ( Syncerus caffer ) for a leave‐one‐out approach. Points represent the mean relative error (%) for one of 50 iterations (excluding a different individual each time), with its 95% confidence intervals. Horizontal dotted lines indicate the ±5% error threshold. Blue points fall within this threshold; red points fall outside of it. Figure S4:2 Relative error (%) in home range area estimates across different sampling durations for simulated Mongolian gazelles ( Procapra gutturosa ) for a leave‐one‐out approach. Horizontal dotted lines indicate the ±5% error threshold. Blue points fall within this threshold; red points fall outside of it. Figure S4:3 Relative error (%) in speed & distance estimates across different sampling intervals for simulated African buffalos ( Syncerus caffer ) for a leave‐one‐out approach. Points represent the mean relative error (%) for one of 50 iterations (excluding a different individual each time), with its 95% confidence intervals. Horizontal dotted lines indicate the ±5% error threshold. Blue points fall within this threshold; red points fall outside of it. [file ECE3-16-e73755-s001.pdf]

Too few, too many, or just right? Optimizing sample sizes for population-level inferences in animal tracking projects

S4. Leave-one out approach

Home range estimation

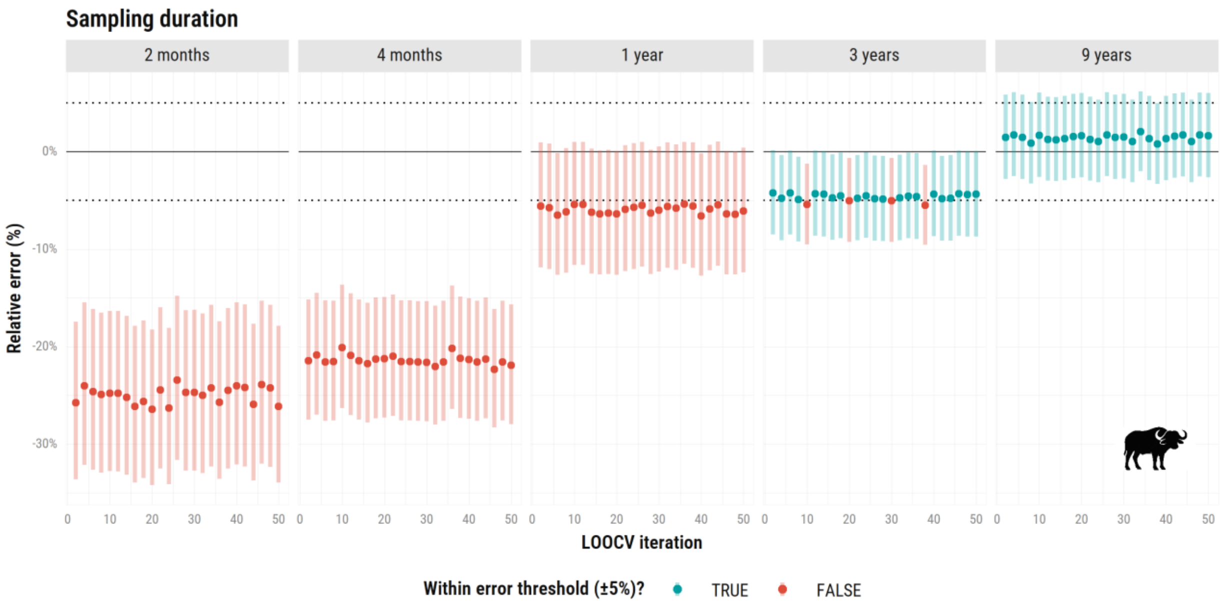

Figure S4.1. Relative error (%) in home range area estimates across different sampling durations for simulated African buffalos (*Syncerus caffer*) for a leave-one-out approach. Points represent the mean relative error (%) for one of 50 iterations (excluding a different individual each time), with its 95% confidence intervals. Horizontal dotted lines indicate the ±5% error threshold. Blue points fall within this threshold; red points fall outside of it.

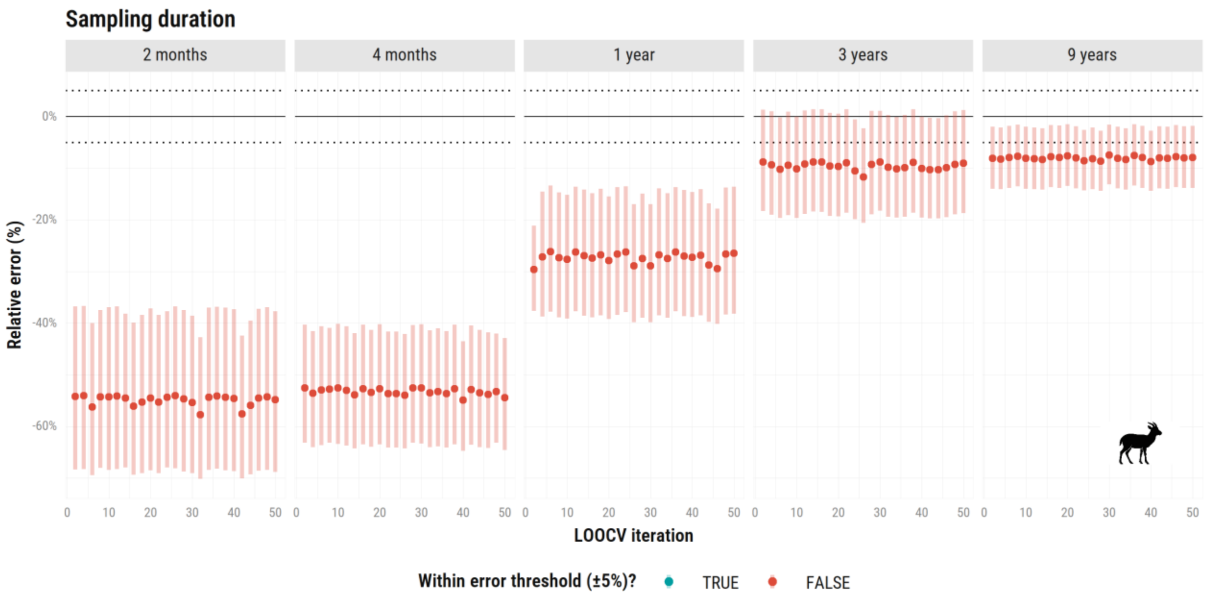

Figure S4.2. Relative error (%) in home range area estimates across different sampling durations for simulated Mongolian gazelles (*Procapra gutturosa*) for a leave-one-out approach. Points represent the mean relative error (%) for one of 50 iterations (excluding a different individual each time), with its 95% confidence intervals. Horizontal dotted lines indicate the ±5% error threshold. Blue points fall within this threshold; red points fall outside of it.

Speed & distance estimation

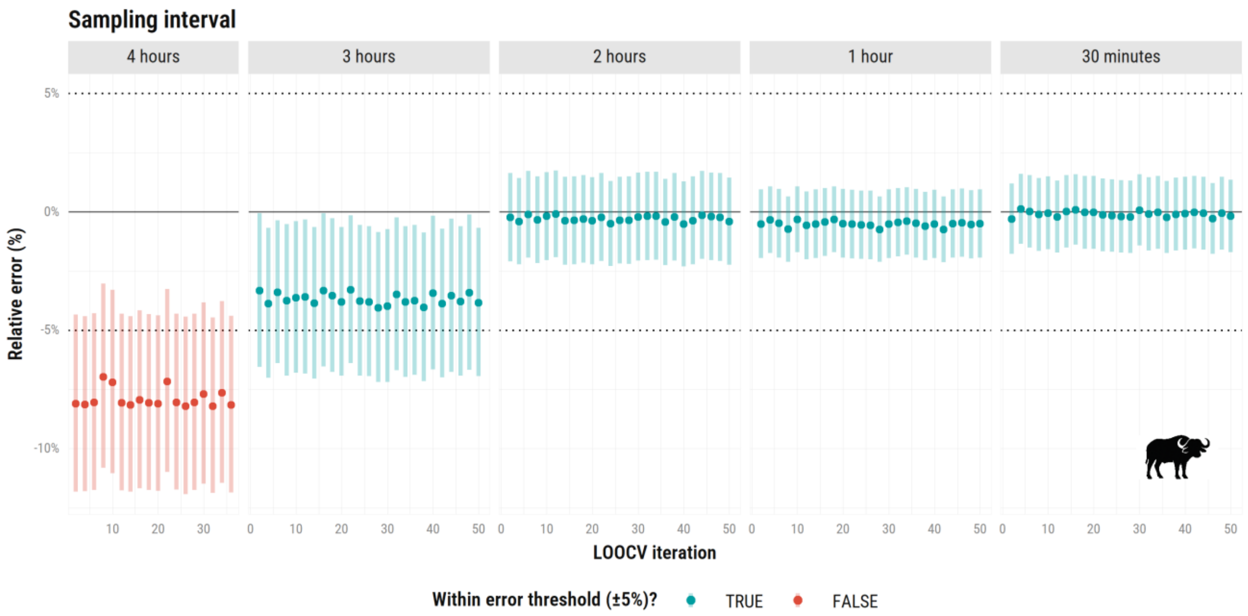

Figure S4.3. Relative error (%) in speed & distance estimates across different sampling intervals for simulated African buffalos (*Syncerus caffer*) for a leave-one-out approach. Points represent the mean relative error (%) for one of 50 iterations (excluding a different individual each time), with its 95% confidence intervals. Horizontal dotted lines indicate the ±5% error threshold. Blue points fall within this threshold; red points fall outside of it.

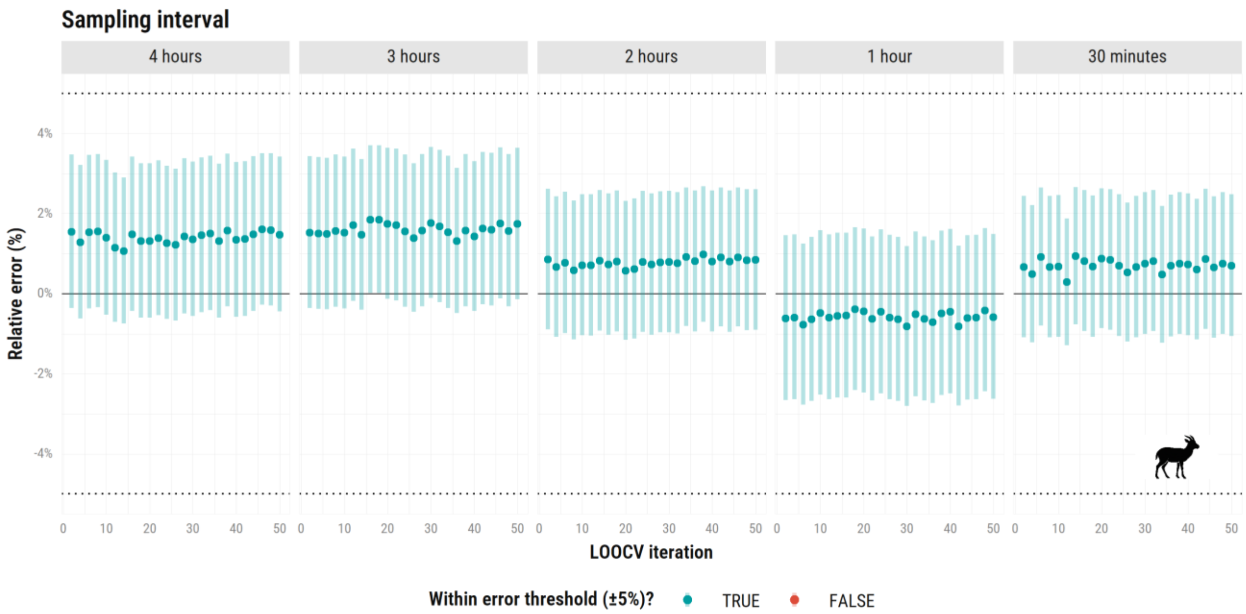

Figure S4.4. Relative error (%) in speed & distance estimates across different sampling intervals for simulated Mongolian gazelles (*Procapra gutturosa*) for a leave-one-out approach. Points represent the mean relative error (%) for one of 50 iterations (excluding a different individual each time), with its 95% confidence intervals. Horizontal dotted lines indicate the ±5% error threshold. Blue points fall within this threshold; red points fall outside of it.
